# Epigenetic modulations in cancer: predictive biomarkers and potential targets for overcoming the resistance to topoisomerase I inhibitors

**DOI:** 10.1080/07853890.2023.2203946

**Published:** 2023-04-24

**Authors:** Moustafa M. Madkour, Wafaa S. Ramadan, Ekram Saleh, Raafat El-Awady

**Affiliations:** a Sharjah Institute for Medical Research, University of Sharjah, Sharjah, United Arab Emirates; b College of Pharmacy, University of Sharjah, Sharjah, United Arab Emirates; c Clinical Biochemistry and Molecular Biology Unit, Cancer Biology Department, National Cancer Institute, Cairo University, Cairo, Egypt

**Keywords:** Cancer, DNA methylation, epigenetic, histone modifications, noncoding RNAs, resistance, Top I, Top I inhibitors

## Abstract

**Introduction:**

Altered epigenetic map is frequently observed in cancer and recent investigations have demonstrated a pertinent role of epigenetic modifications in the response to many anticancer drugs including the DNA damaging agents. Topoisomerase I (Top I) is a well-known nuclear enzyme that is critical for DNA function and cell survival and its inhibition causes DNA strand breaks and cell cycle arrest. Inhibitors of human Top I have proven to be a prosperous chemotherapeutic treatment for a vast number of cancer patients. While the treatment is efficacious in many cases, resistance and altered cellular response remain major therapeutic issues.

**Areas covered:**

This review highlights the evidence available till date on the influence of different epigenetic modifications on the response to Top I inhibitors as well as the implications of targeting epigenetic alterations for improving the efficacy and safety of Top I inhibitors.

**Expert opinion:**

The field of epigenetic research is steadily growing. With its assistance, we could gain better understanding on how drug response and resistance work. Epigenetics can evolve as possible biomarkers and predictors of response to many medications including Top I inhibitors, and could have significant clinical implications that necessitate deeper attention.

**HIGHLIGHTS**
- Epigenetic alterations, including DNA methylation and histone modifications, play a pertinent role in the response to several anticancer treatments, including DNA damaging agents like Top I inhibitors.
- Although camptothecin derivatives are used clinically as Top I inhibitors for management of cancer, certain types of cancer have inherent and or acquired resistance that limit the curative potential of them.
- Epigenetic modifications like DNA hypomethylation can either increase or decrease sensitivity to Top I inhibitors by different mechanisms.
- The combination of Top I inhibitors with the inhibitors of histone modifying enzymes can result in enhanced cytotoxic effects and sensitization of resistant cells to Top I inhibitors.
- MicroRNAs were found to directly influence the expression of Top I and other proteins in cancer cells resulting in positive or negative alteration of the response to Top I inhibitors.
- lncRNAs and their genetic polymorphisms have been found to be associated with Top I function and the response to its inhibitors.
- Clinical trials of epigenetic drugs in combination with Top I inhibitors are plentiful and some of them showed potentially promising outcomes.

## 1. Introduction

Chromatin is a dynamic structure that controls the interaction of regulatory factors with the genetic material [1]. DNA and histone proteins comprise the chromatin, which can be remodelled by epigenetic mechanisms into a tightly condensed or an open state conformation, to influence gene expression. These epigenetic mechanisms regulate the interaction between DNA and histone proteins, which is accomplished by changes in DNA methylation, histone post-translational modifications and by the processes regulated by the non-coding RNAs [2]. These changes orchestrate the expression of genes through modulating the accessibility of transcription factors or DNA binding proteins to DNA [3].

Methylation of cytosine nucleotides in the context of CpG dinucleotides is the most commonly studied epigenetic mechanism that is mediated by DNA methyltransferase (DNMT) enzymes [3,4]. Changes in DNA methylation have a considerable effect on gene expression; hypomethylation results in amplifying the gene expression while hypermethylation induces gene silencing [5–10]. In addition, diverse post-translational modifications to histone proteins such as methylation, acetylation or phosphorylation have been reported to promote the relaxation of chromatin structure to activate gene transcription or to promote the chromatin coiling that facilitates transcriptional repression [10]. Recently, there is a growing realisation of the regulatory role of non-coding RNA in epigenetic modulation. Among them are miRNAs, piRNAs, endogenous siRNAs, and long non-coding RNAs are the most abundant regulatory RNAs embroiled in regulating gene expression [11].

The stable maintenance of epigenetic landscapes within the eukaryotic genome, established during DNA replication, transcription and repair, is critical for the maintenance of chromatin integrity. Over the last years, the disruption of epigenetic machinery has been observed in cancer as abnormal patterns of DNA methylation and histone posttranslational modifications. Several reports have suggested that these epigenetic alterations could be involved in the individual patient variation in response to anticancer drugs and in drug resistance [12]. Some tumor suppressor genes (TSGs) such as *RASSF10*, *SIX3*, *CDKN2A*, *PTEN*, *TIMPS*, *DAPK*, *LY6K* and *SLC34A2* display altered expression in tumor cells resulting from epigenetic modifications [13]. Also, several efflux transporters show elevated expression that correlates with promoter hypomethylation in certain tumor cells leading to drug resistance phenotype [14,15]. Furthermore, one of the foremost responses to DNA damage is epigenetic alteration and chromatin remodeling which, in coordination with other pathways, determine the ultimate cellular response to DNA damage. They are known to affect the expression of DNA repair genes and their accessibility to the sites of DNA damage. Thus, modulation of such pathways has been determined to play a significant role in the repair of DNA strand breaks [16–18]. Therefore, epigenetic modifications could be essential for the response to many anticancer drugs, especially the DNA damaging agents.

Topoisomerase I-targeting drugs represent one of the main families of DNA damaging drugs and was found to have a great anticancer potential. In this review, we will highlight the role of epigenetics in mediating the response of cancer cells to topoisomerase I inhibitors and its clinical relevance.

## 2. Topoisomerase I inhibitors and drug resistance

### 2.1. Topoisomerase I inhibitors

Topoisomerases (Tops) are enzymes that solve DNA topological problems such as supercoils and overwinds resulting from detachment of the double helix’s complementary strands of the DNA during replication, transcription and recombination. There are two well-characterized types of Tops, which are Top I and Top II. Type I Tops cut a single strand of DNA to relax the torsional stresses before religation. On the other hand, type II Tops fissure both DNA strands to expedite the passage of an intact duplex through the gap before reconciling the DNA backbone bonds. In all contexts, Tops modify the topological state of nucleic acids by forming topoisomerase cleavage complexes (TOPccs) [19–25]. Indeed, Tops are proved targets of a wide spectrum of anticancer agents due to their central role in DNA metabolism, particularly in proliferating cells. For example, the camptothecin (CPT) derivatives such as irinotecan and topotecan selectively target Top I [26], while etoposide and anthracyclines are well recognized in targeting Top II [27].

The mechanism of action of Top I inhibitors involve either stabilizing TOPccs (termed poisons) or otherwise inhibiting Top’s catalytic activity (termed catalytic inhibitors), causing DNA damage [28,29]. The Top I-DNA covalent complex can be snared by Top I poisons like CPTs, which then turn the enzyme into a cytotoxic covalently bonded protein adduct on DNA. On the other hand, Top I catalytic inhibitors act on any other step in the catalytic cycle and can prevent the formation of the cleavage complexes. Due to this, DNA replication is inhibited, and double strand breaks (DSBs) are produced due to the collapse of the replication forks [28]. Along with CPTs, the anticancer agents indenoisoquinolines and indolocarbazoles are likewise classified as Top I poisons [30–33]. Currently, Top I poisons such as the CPT derivatives topotecan and irinotecan are utilized in the clinic for the treatment of ovarian, pancreatic, small cell lung, and colorectal cancers, while all catalytic inhibitors like betulinic acid and CYB-L1 are still in the development stage [34–43].

### 2.2. Mechanisms of resistance to Topoisomerase I inhibitors

**Figure 1.**
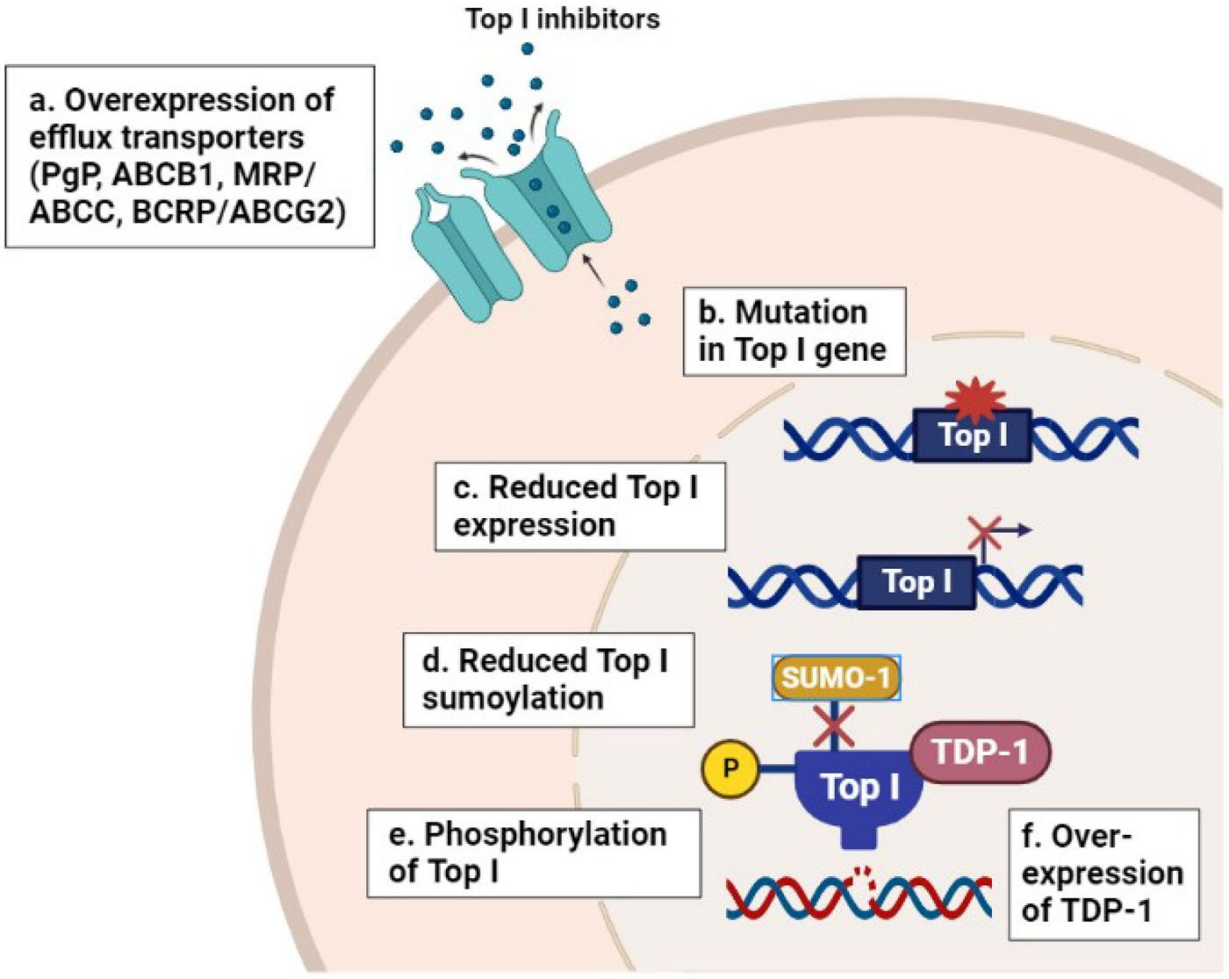
Resistance mechanisms to Top I inhibitors in cancer. (a) Overexpression of efflux transporters (PgP, ABCB1, MRP, ABCC and BCRP) can reduce the cellular abundance of Top I inhibitors. (b–e) Mutations in *Top I* gene, reduced Top I expression and phosphorylation levels and reduced sumoylation of Top I enzyme itself can decrease the affinity of Top I inhibitors to their target. (f) Overexpression of tyrosyl-DNA phosphodiesterase 1 (TDP-1) can increase the rate of cleavage of the covalent linkage between stabilized Top1 with DNA and reverses the formation of cleavage complex. All these events lead to the development of resistance to Top I inhibitors. PgP: P-glycoprotein; ABCB1: ATP Binding Cassette Subfamily B Member 1; MRP/ABCC: multidrug resistance related protein; BCRP/ABCG2: breast cancer resistance protein; Top I: topoisomerase I; TDP-1: tyrosyl-DNA phosphodiesterase 1.

When cells are exposed to Top I inhibitors, an orchestrated sequence of events occurs including stabilization of the cleavage complexes followed by processing of stabilized cleavage complexes into deadly DNA lesions (i.e. DSBs), leading to the induction of stress-related signaling pathways (e.g. cell-cycle arrest, DNA repair and apoptosis) [44]. It is well reported that the response to Top I inhibitors is regulated by multiple mechanisms including the expression of efflux transporters such as P-glycoprotein (PgP, ABCB1), multidrug resistance related proteins (MRP, ABCC), and breast cancer resistance protein (BCRP or ABCG2) [45–47]. In addition to the mutations in Top I gene, that are frequently observed in CPT-resistant cell lines [48,49], and expression level of Top I in which its reduction leads to the development of resistance to CPT *in vitro* and *in vivo* [50]. Furthermore, the phosphorylation of Top I by protein kinase CK2 can alter the cellular response to CPT [51]. CPT resistance has been linked with alterations in DNA repair pathways of Top I cleavage complex that caused by the overexpression of tyrosyl-DNA phosphodiesterase 1 (TDP-1), activation of AKT and dysfunction of p53 [52]. Moreover, regulation of Top I function is accomplished by post-translational modification including SUMOylation, which affect the formation of cleavage complexes induced by CPT [53]. Figure 1 summarizes some resistance mechanisms to Top I inhibitors.

Despite the fact that CPTs have the capacity to inhibit one of the most important DNA replication enzymes, they are not highly active as single agents. According to previous reports, CPT had a 10% to 15% success rate in managing certain cancer indications [54], and many patients developed tumor relapses within two years. Previous studies demonstrated that CPTs can be inactivated by hydrolysis of its active lactone form. Additionally, although CPTs freely enter cells by passive diffusion, the intracellular concentration of these drugs is decreased by the efflux pumps. Up to date, the mechanisms of resistance are still emerging, likely involving pharmacological (pharmacokinetics and pharmacodynamics), tumor and genetic related factors and more attention is being paid to epigenetic changes to address new factors involved in resistance to Top I targeted therapy [32,55–60].

## 3. Epigenetic modifications and the response of cancer cells to Topoisomerase I inhibitors

### 3.1. DNA methylation

**Figure 2.**
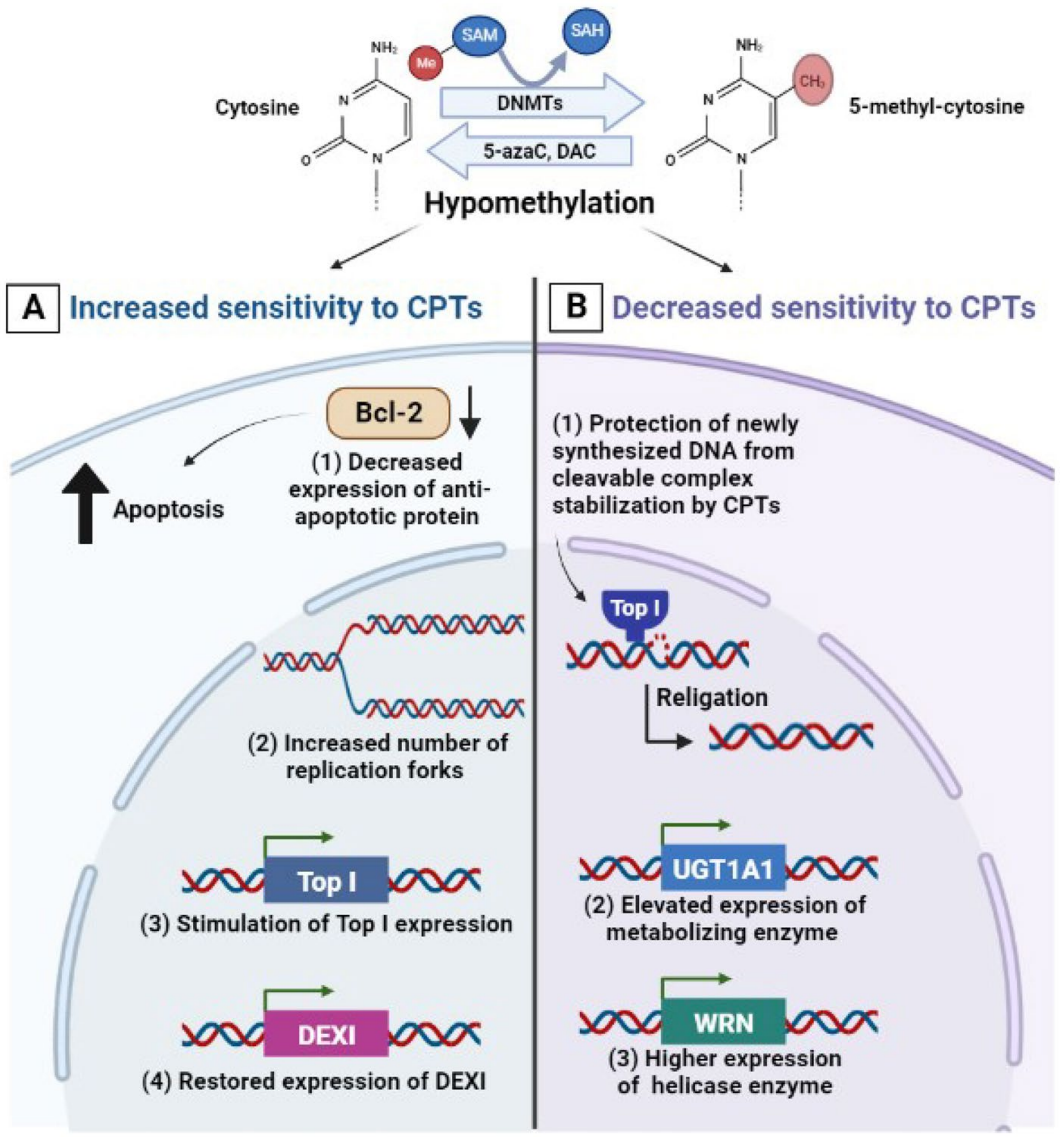
Effect of DNA hypomethylation on the response of cancer cells to CPTs. (A) mechanisms by which hypomethylation can increase sensitivity to CPTs including: decreased expression of Bcl-2 anti-apoptotic protein, increased number of replication forks in early S phase, demethylation of the Top I promoter or indirect stimulation of Top I expression and restored expression of DEXI protein. (B) Mechanisms by which hypomethylation can decrease sensitivity to CPTs including protection of newly synthesized DNA from cleavage complex stabilization and DNA fragmentation, elevated expression of UGT1A1 metabolizing enzyme which is responsible for irinotecan’s inactivation and elevated expression of WRN helicase enzyme.

DNA methylation is known to affect the interaction with certain DNA-binding proteins including DNA Tops. The role of DNA methylation in controlling the response of cancer cells to drugs including Top I inhibitors was identified by the use of the hypomethylating cytidine analogue 5-azacytidine (5-azaC). The pre-treatment of Chinese hamster ovary cells with 5-azaC was demonstrated to increase their sensitivity to CPT and to result in a strong synergistic effect on chromosomal damage. This could be premised on the idea that changing chromosome replication timing after DNA hypomethylation increases the number of replication forks in early S phase, which subsequently increases the likelihood of collision between a blocked DNA-Top I-CPT cleavage complex and the replication fork [61]. In addition, the cytotoxicity of irinotecan was demonstrated to be increased by 5-azaC in colorectal cancer cells *via* at least one of the following mechanisms: (a) demethylation of the Top I promoter, (b) indirect stimulation of Top I expression, and (c) amendment of cell cycle progression and/or apoptosis following DNA damage [62,63] (Figure 2(A)). Interestingly, the combination of 5-azaC and irinotecan resulted in a synergistic response with considerable improvement in survival and tumor regression in human colon cancer xenograft mice [64,65]. In pheochromocytoma/paraganglioma, the intermittent coadministration of 5-azaC also increased the efficacy of low doses of CPT and other Top I inhibitors in *in vitro* and *in vivo* settings [66].

Indeed, another hypomethylating cytidine analogue, 5-aza-2′-deoxycytidine (DAC), enhanced the anticancer efficacy of CPT-11, the prodrug of irinotecan, additively and its active metabolite, 7-ethyl-10-hydroxycamptothecin (SN-38), synergistically. The extent to which DAC potentiates CPT-11 or SN-38 might be dependent on the expression level of anti-apoptotic Bcl-2 protein in human colorectal cancer cells, as the higher intracellular protein levels of Bcl-2 were shown to be associated with the resistance of cancer cells to CPT-11 and SN-38 [67–70]. Treatment with DAC before irinotecan significantly improved tumor suppression in a xenograft model with OCUM2 M/SN38 irinotecan-resistant gastric cancer cells. Irinotecan showed robust tumor suppression effects after epigenetic priming and caused massive cell loss with fibrosis, inflammation and induced cellular enlargement. These findings indicate that priming with DAC increased the cytotoxic effects of irinotecan *in vitro* and *in vivo* [71,72]. Furthermore, inhibition of DNMTs by DAC followed by CPT administration resulted in a significant suppression of proliferation and induction of apoptosis in hepatocellular carcinoma cells (SMMC-7721) [73]. On the contrary, the hypomethylation induced by DAC diminished the capability of CPT to stabilize the enzyme-DNA cleavage complexes that are responsible for CPT’s-induced DNA damage in cultured Chinese hamster ovary cells. It was suggested that hypomethylation induced by the incorporation of DAC into newly synthesized DNA in the place of cytosine may protect DNA from cleavage complex stabilization and DNA fragmentation which is induced by CPT in a dose-dependent manner as compared with CPT alone [74] (Figure 2(B)).

Current evidence indicates that DNA methylation has a central role in drug metabolism through regulating the expression of drug-metabolizing enzymes which affects the metabolic process of anticancer drugs and contributes to individual variations in drug response. It was demonstrated that DNA methylation suppresses the expression of *UGT1A1*, a metabolizing enzyme involved in the inactivation of irinotecan’s active metabolite SN-38, contributing to the level of tumoral inactivation of SN-38. In this regard, *UGT1A1* expression was significantly elevated in colorectal cancer cell lines treated with DAC (demethylating agent) and trichostatin A (histone deacetylase inhibitor), resulting in increasing the production of SN-38 inactive glucuronide (Figure 2(B)). These findings reveal that the demethylation at *UGT1A1* promoter site is associated with tumor cells having a significantly higher SN-38 glucuronidation capability and this could contribute to lower response of cancer cells to the drug [75].

The aberrant DNA hypermethylation is frequently detected in cancer cells which resulted in silencing the expression of certain genes such as Werner (*WRN*), a gene coding for a DNA helicase that helps in maintaining genomic integrity. *WRN* expression was thought to influence the sensitivity of cancer cells to DNA Top I inhibitors. For instance, cervical cancer cells were more sensitive to irinotecan after the downregulation of *WRN*. In line with this, the susceptibility of cervical cancer cells to irinotecan was likewise boosted by *WRN* DNA hypermethylation. Treatment with a demethylating agent restored the *WRN* expression in cervical cancer cells and decreased their sensitivity to irinotecan (Figure 2(B)). These findings imply that aberrant *WRN* methylation plays a key role in the sensitivity of cervical cancer cells to CPTs [76]. On the other hand, methylation of dexamethasone-induced protein (*DEXI*), a glucocorticoid-induced protein-coding gene, is involved in facilitating CPT resistance *via* inhibition of apoptosis. Restoring *DEXI* expression *via* exogenous induction or treatment with DAC improved the susceptibility to CPT. Interestingly, following irinotecan-based chemotherapy, colorectal cancer patients with negative *DEXI* methylation status had better overall survival than patients with positive *DEXI* methylation status. This indicates that *DEXI* methylation is linked to irinotecan’s poor response and efficacy, implying that *DEXI* could be utilized as a potent therapeutic target as well as its methylation status as an epigenetic biomarker to identify individuals who will benefit more from chemotherapy based on CPTs [77].

The aforementioned data indicate that DNA hypomethylation can either increase or decrease sensitivity to Top I inhibitors (Figure 2). This could depend on a number of variables, including the location, the position of the methyl residues with respect to the cleavage site, the type of genes affected and the degree of DNA methylation may correlate with the ability of Top I inhibitors to stabilize Top I-DNA complexes and to facilitate DNA cleavage. Given that DNA methylation affects both the pattern of replication and the expression of various genes, this may present a chance to improve chemotherapy strategies that initially aim to raise the number of replication forks and stimulate the expression of the enzyme Top I in targeted cells by hypomethylating agents. However, as mentioned previously, hypomethylation in some cases may protect against cleavage complex stabilization and DNA DSBs induced by Top I poisons and this highlights the importance of understanding the relationship between the pattern of DNA methylation, Top I activity and genomic instability.

### 3.2. Histone modifications

#### 3.2.1. Histone acetylation

Changes in histone modification patterns such as acetylation can be utilized as promising biomarkers when considering resistance to anticancer drugs including Top I inhibitors. Histone acetyltransferases (HATs) and histone deacetylases (HDACs) are epigenetic enzymes that control the acetylation level of histone proteins, and their activities are known to be dysregulated in cancer. For decades, HDACs have been considered promising targets in cancer therapy and several HDAC inhibitors have been approved by FDA for the treatment of certain types of cancer [78,79]. The FDA-approved pan-HDAC inhibitor vorinostat was found to enhance the cytotoxicity of Top I inhibitors *in vitro* [80,81]. Although it was reported that vorinostat did not enhance DNA DSBs on its own, however, when combined with irinotecan, vorinostat boosts irinotecan’s cytotoxicity and potentiates DNA damage by increasing the number of early irinotecan-induced DNA DSBs and inhibiting their subsequent repair [80]. In addition, the combination of vorinostat and topotecan resulted in significant colony formation inhibition in small cell lung cancer (SCLC) cells. Indeed, the use of equipotent dosages of both vorinostat/topotecan and vorinostat/CPT combinations as well as low doses of vorinostat and higher doses of Top I inhibitor, showed a high synergistic effect in sensitive and resistant cell lines. It was shown that vorinostat does not directly influence Top I activity or content, but rather enhances the action of Top I inhibitors by increasing the amount of Top I/DNA cleavage complexes [81].

The combination of MGCD0103 (moncetinostat), a selective HDAC inhibitor, with topotecan was found to have an additive effect leading to apoptosis and caspase activation versus either agent alone against SCLC cell lines [82]. The synergistic increase in caspase-3/7 activity and apoptosis induction has also been reported upon the combination of the pan HDAC inhibitor panobinostat and topotecan in cervical cancer cells. However, it was shown that the synergistic interaction between panobinostat and Top I inhibitors was schedule-dependent. Pretreatment with topotecan for 24 h prior to panobinostat addition was synergistic, albeit to a lesser extent than the concurrent dosing, while antagonistic effect was seen with the pretreatment with panobinostat for 24 h before topotecan. The synergistic effect of panobinostat and topotecan combination was found to be exerted by generating reactive oxygen species (ROS) and activating the mitochondrial intrinsic apoptotic pathway. Moreover, this combination treatment effectively inhibited the migratory capabilities of cervical cancer cells [83,84]. The enhanced level of DSBs and the subsequent apoptosis induction was also indicated upon the combination of valproic acid and the Top I inhibitor karenitecin in mouse xenografts [85]. Increased DNA DSB repair rate was found to be linked to changes in the chromatin acetylation landscape, in particular, histone H4K16 acetylation (H4K16ac). Downregulation of H4K16ac was observed upon the treatment of colorectal cancer resistant cells with irinotecan. Subsequent treatment of irinotecan-resistant cells with HDAC inhibitors, including trichostatin A or panobinostat, effectively enhanced H4K16 acetylation and sensitized resistant cells to CPT therapy [86].

One of the main concerns of using Top I inhibitors in anticancer combination therapy is the risk of drug-drug interactions, which may lead to reduced efficiency or severe toxicity to normal cells. Drug metabolism through glucuronidation was reported as an important source of drug-drug interaction when Top I inhibitors were combined with HDAC inhibitors. Glucuronidation *via* UDP-glucuronosyltransferase enzymes (UGTs) has been shown to deactivate Top I inhibitors (such as SN-38) and HDAC inhibitors such as belinostat, vorinostat and panobinostat. Thus, they all compete for the deactivation by UGTs. It was reported that belinostat only, but not vorinostat or panobinostat, inhibited SN-38 glucuronidation *via* inhibiting the activity of UGT1A1. As the concentration of belinostat increased, the rate of SN-38 glucuronide (SN-38G) formation decreased dose-dependently, indicating a non-competitive increase in the inhibition of SN-38 glucuronidation. This emphasizes the potential clinical significance of drug-drug interaction between irinotecan and belinostat, since many individuals could be at risk of experiencing severe toxicity if the two drugs are administrated together [87].

Recent advances in drug design and discovery led to the development of dual inhibitors, which hit multiple targets with a single molecule, to overcome the limitations of combination therapies. Numerous studies reported the discovery of dual inhibitors simultaneously targeting Top I and HDACs. Novel dual acting HDAC-Top I inhibitors were generated by covalently merging SAHA-like HDAC inhibitor to the CPT framework. These hybrid molecules effectively inhibited the proliferation of numerous cancer cell lines and showed promise as potent anticancer agents with the potential to significantly arrest tumor development by blocking two key enzymes [88,89]. Another selected multivalent agent also containing a CPT and a SAHA-like template showed a broad spectrum of antiproliferative activity, with IC50 values in the nanomolar range, on a series of human solid tumor, hematologic, and mesothelioma cell lines. Interestingly, in comparison to SAHA and topotecan, the new hybrid molecule demonstrated higher HDAC inhibitory potency, improved anticancer activity, and extremely excellent tolerability *in vivo*. Similarly, another single dual-acting active molecule comprising CPT and HDAC inhibitor psammaplin A, displayed a significant anticancer activity and a very good tolerability. As a result, these dual inhibitors can be utilized to treat tumors that are sensitive to CPT derivatives and/or HDAC inhibitors, with good tolerability and low toxicity [90,91].

Despite the vast number of studies about sensitization to Top I inhibitors through HDAC inhibition, the inhibition of HATs was also reported. Gene deletion of multiple HATs resulted in higher DNA damage levels and significant defects in resistance to CPT [92–97]. It was found that NuA4 acetyltransferase and histone H4 acetylation promote the repair of broken DNA replication forks and are involved in mediating CPT resistance [98,99]. In line with this, the downregulation of NuA4 subunits sensitized resistant cells to CPT [98]. Moreover, inhibition of HAT by a spermidine S-substituted coenzyme A (CoA) inhibitor was also associated with enhanced cellular sensitivity to CPT, indicating the vital role of HATs in the response to Top I inhibitors [100].

Chromatin readers known as bromodomain and extraterminal domain (BET) protein family can interact with acetylated histone tails to recruit chromatin remodelling and transcription proteins to DNA. It has been unearthed that BET inhibition synergizes with several drugs that target DNA damage signalling and repair, such as Top I inhibitors. The novel BET inhibitor JQ1 can synergize with CPT, exhibiting antiproliferative effects and resulted in enhancing the *in vivo* susceptibility of tumors to CPT without causing toxicity [101–104]. Another BET inhibitor, OTX015, potentiated the anticancer activity of CPT and LMP400, a noncamptothecin Top I inhibitor, in castration-resistant prostate cancer patient-derived explants and xenograft models by disrupting the DNA replication fork stability during cellular division [105]. Furthermore, in glioblastoma xenografts, OTX015 showed additive to synergistic efficacy when paired with irinotecan [106]. The depletion or disruption of yeast Bromodomain Factor 1 or Bromodomain Factor 2 (its human counterpart TAF1) was found to increase susceptibility to CPT through inhibiting the DNA end resection and repair [107,108].

#### 3.2.2. Histone methylation

Histone methylation is the dynamic addition of one, two, or three methyl groups to specific amino acids within a histone protein. Nearly all biological processes, including DNA repair, cell cycle, stress response, transcription, development, differentiation, and aging, have been shown to be regulated by histone methylation [109]. Since abnormal histone methylation has been reported to play a causal role in tumorigenesis, it can be linked to anticancer-related drug responses [110]. Histone lysine demethylases (KDMs) are enzymes that catalyze the removal of methyl group from lysine and arginine residues on histone tails and were found to play critical roles in oncogenesis [111]. Addition of the KDM inhibitor 17-DMAG to the clinically tested combination vincristine and irinotecan significantly improved the efficacy of this combination, indicating that targeting KDM may serve as a useful approach for enhancing the response to anticancer drugs like Top I inhibitors [112].

**Figure 3.**
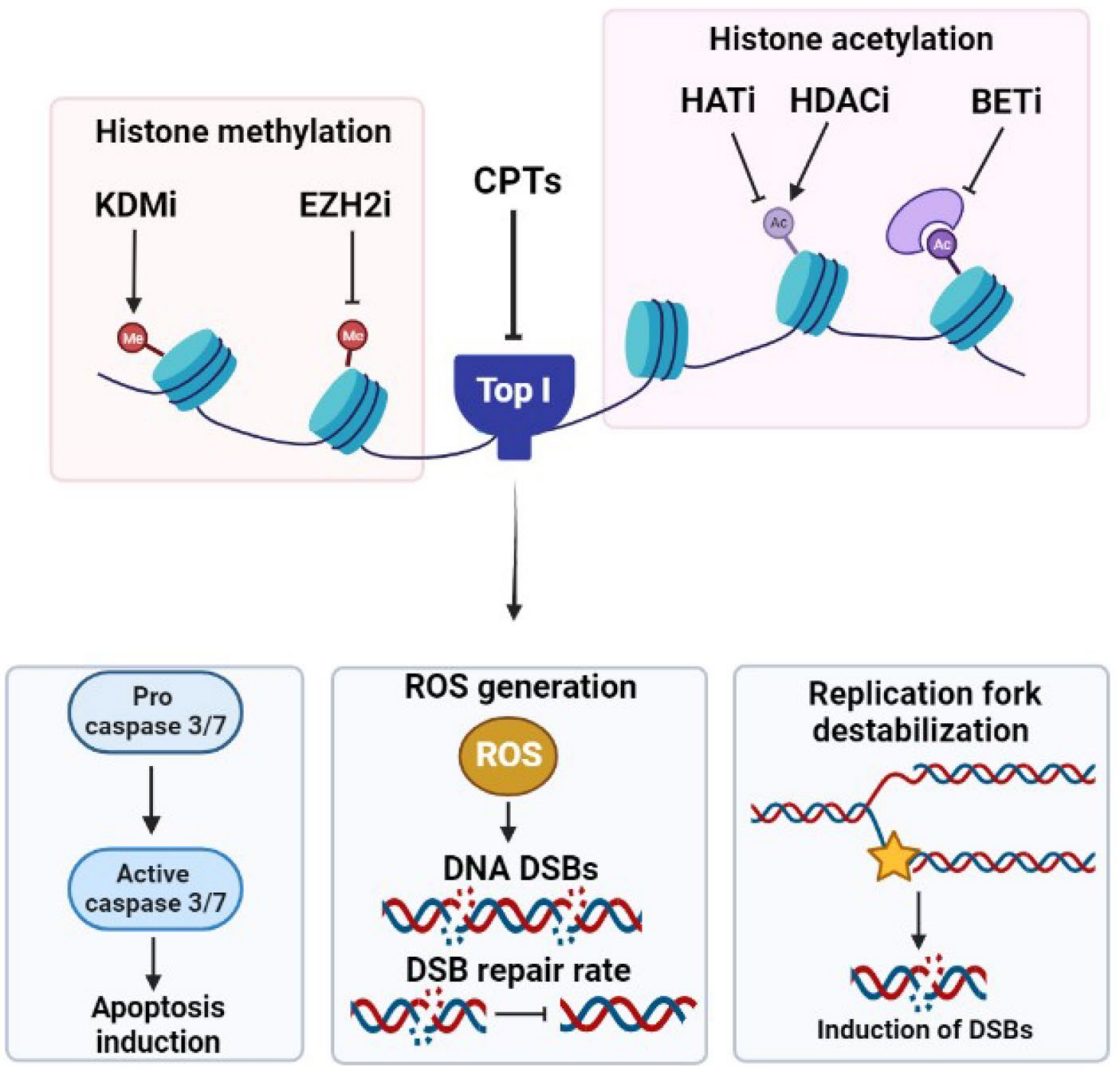
Effect of targeting histone modifying enzymes on cellular response to CPTs. Gene deletion or inhibition of HATs, inhibition of histone deacetylation by HDACi or inhibiting BET, KDMs and EZH2 result in higher DNA damage levels, ROS generation, increase in caspase 3/7 activity and apoptosis induction. KDMi: histone lysine demethylase inhibitor; EZH2i: histone-lysine N-methyltransferase enzyme enhancer of zeste homolog 2 inhibitor; CPTs: camptothecins; Top I: topoisomerase I; HATi: histone acetyltransferase inhibitor; HDACi: histone deacetylase inhibitor; BETi: bromodomain and extraterminal domain inhibitor; DSBs: double strand breaks; ROS: reactive oxygen species.

The histone-lysine N-methyltransferase enzyme enhancer of zeste homolog 2 (EZH2) catalyzes the addition of methyl groups to histones leading to gene silencing. Mutations in EZH2 have been associated with numerous malignancies [113,114]. Inhibition of EZH2 is demonstrated to sensitize castration-resistant prostate cancer cells to CPT treatment both *in vitro* and in mice models. Additionally, CPT treatment results in decreasing the EZH2 expression and enhancing death of cancer cells [115]. The combination of the EZH2 inhibitor EPZ011989 and irinotecan significantly improved the survival outcomes of female sarcoma mice models [116]. These findings suggest that inhibiting EZH2 would be an essential strategy for enhancing the activity of Top I inhibitors. Figure 3 summarizes the effects of histone modifying drugs on the response to Top I inhibitors.

### 3.3. Noncoding RNAs

While DNA methylation and histone modifications were extensively studied, it is evident that RNA-mediated processes require attention as well since they play key roles in a variety of biological processes including regulation of mRNA expression, chromatin remodelling, post-transcriptional regulation, disease pathogenesis and other epigenetic-related functions. Non-coding RNAs (ncRNAs) comprise more than 90% of the transcripts in our cells and they don’t have protein coding roles [117]. Regulatory ncRNAs are assorted into microRNAs (miRNAs), Piwi-interacting RNAs (piRNAs), enhancer RNAs (eRNAs), small interfering RNAs (siRNAs) and long non-coding RNAs (lncRNAs). Several of these have been shown to play a role in controlling gene expression, including modulation of the binding of some proteins to DNA such as Top I [118–121].

#### 3.3.1. MicroRNAs

**Figure 4.**
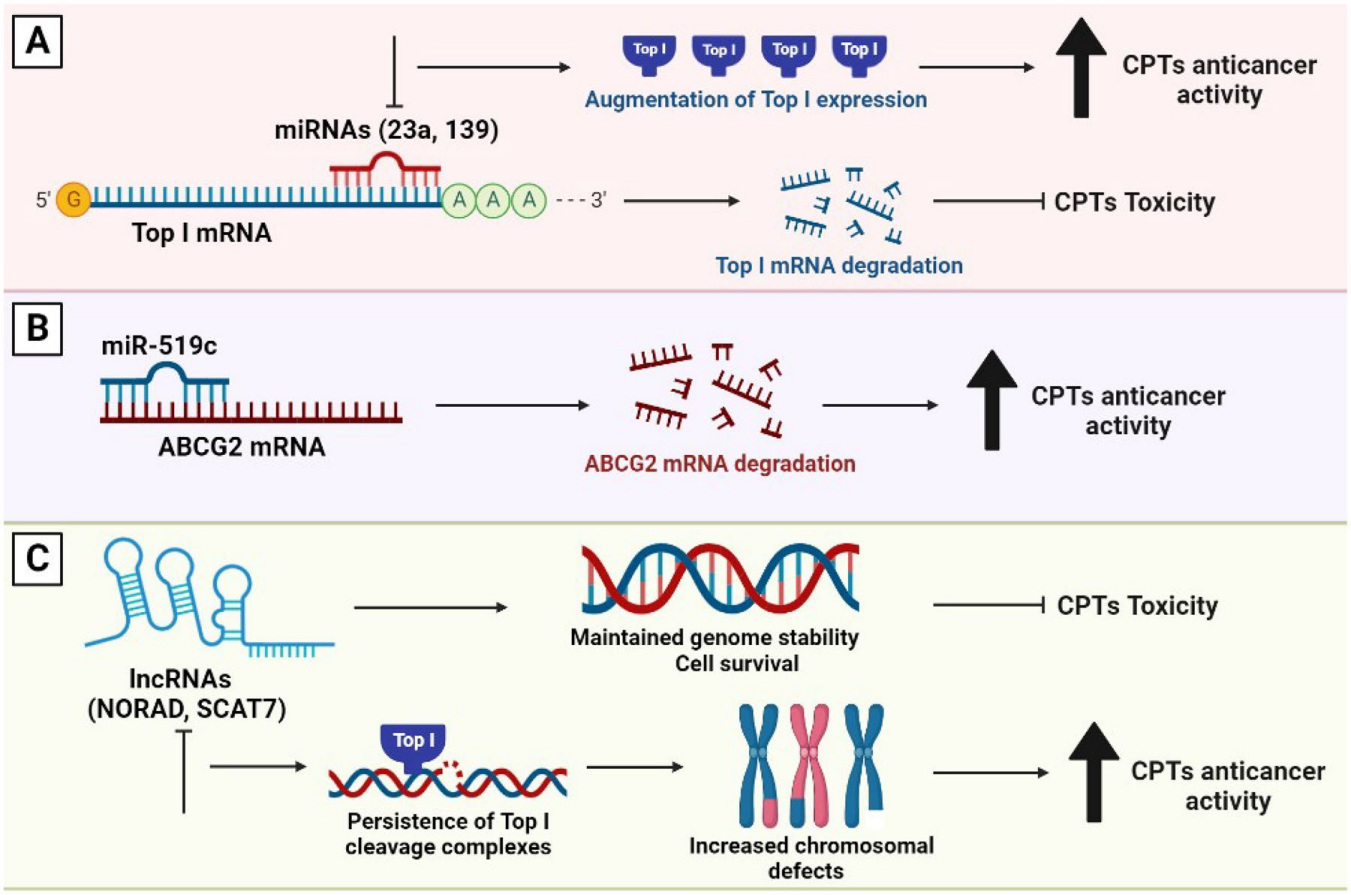
The involvement of miRNAs and lncRNAs in the response to Top I inhibitors. (A) miR-23a and miR-139 inhibit Top I expression. Thus, curbing their expression will in turn augment the enzyme’s expression. (B) Higher levels of miR-519c can lead to decreased ABCG2 expression leading to higher sensitivity to CPTs. (C) lncRNAs participate in maintaining genomic stability, cell survival and higher resistance to CPTs. Absence of lncRNAs can lead to persisitence of Top I cleavage complexes, increased chromosomal defects and better response to CPTs.

MicroRNAs (miRNAs) are a class of small ncRNAs, which function in post-transcriptional regulation of gene expression. They are powerful regulators of various cellular activities including cell growth, differentiation, development and apoptosis. Therefore, they have been linked to many diseases, including cancer [122,123]. Interestingly, miRNAs were found to directly affect Top I expression in cancer cells. For example, miR-23a and miR-139 were found to inhibit Top I expression in hepatocellular carcinoma (HCC). These miRNAs were reported to bind directly to the 3′ untranslated region (UTR) of Top I mRNA and to suppress the expression of the corresponding protein. Thus, the inhibition of miR-23a or miR-139 further augments Top I expression (Figure 4(A)). The fact that forced overexpression of these miRNAs might attenuate the cytotoxicity of Top I poisons through Top I downregulation further demonstrates the link between miRNAs and Top I. These findings indicate that Top I is a direct target of miR-23a and miR-139 [124,125].

In addition to Top I, miRNAs can induce mRNA degradation and translation repression of multidrug resistance-associated ABC transporters such as ABCG2. It was reported that miR-519c is a direct regulator of ABCG2 expression. Low levels of miR-519c can lead to increased ABCG2 levels and vice versa (Figure 4(B)). Indeed, overexpression of ABCG2 *in vitro* has been shown to cause resistance to irinotecan. Therefore, miR-519c and ABCG2 have been suggested as biomarkers for determining the response of colorectal cancer patients to irinotecan-based chemotherapy [126]. Furthermore, overexpression of miR-124 was found to be linked to a decreased DNA repair capacity in cultured cancer cells and increased sensitivity to DNA-damaging antitumor drugs, particularly those causing DNA DSBs (Figure 4(A)). It was shown that after recovery from CPT, cells with higher miR-124 levels showed significantly more residual DNA DSBs, indicating a decreased repair capacity [127].

#### 3.3.2. Long non-coding RNAs

Long non-coding RNAs (lncRNAs) are RNAs longer than 200 nucleotides aberrantly expressed in human cells and play crucial roles in gene regulation. The most well-studied example of lncRNAs is the lncRNA activated by DNA damage (NORAD), which is required for maintaining genome stability. NORAD interacts with RBMX, a component of the DNA-damage response, and promotes the assembly of a ribonucleoprotein complex known as NORAD-activated ribonucleoprotein complex 1 (NARC1). NARC1 complex contains some proteins, including Top I, which are known to enhance genomic stability [128]. In colorectal cancer cells, the knockdown of either RBMX or NORAD increased the incidence of chromosomal segregation errors, decreased replication-fork velocity, and disrupted cell-cycle progression (Figure 4(C)). These events represent phenotypes that are linked to Top I function and could influence the response to its inhibitors [128,129]. Another lncRNA involved in the maintenance of genome integrity is SCAT7 (ELF3-AS1). SCAT7 is essential for cell survival and is increased upon exposure to DNA-damaging drugs such as CPT. Additionally, SCAT7 participates in the proteasome-mediated degradation of Top I and its absence leads to the persistence of TOP1cc that interfere with replication fork progression, resulting in significant intrinsic DNA damage (Figure 4(C)). Therefore, SCAT7 operates as a Top I scaffold by interacting with it and regulating its turnover *via* a ubiquitin-dependent proteasome pathway. Thus, SCAT7 can be utilized as a predictive biomarker for response to Top I inhibitors [130].

Genetic polymorphisms are common among lncRNAs and were found to be associated with cancer risk and variable drug treatment response [131,132]. HOTAIR and MALAT1 are lncRNAs that are coupled with poor cancer prognosis. They have been found to impact drug resistance, apoptosis and cellular proliferation. Neither *rs3200401* MALAT1 nor *rs4759314* HOTAIR polymorphisms were found to be associated with the response to irinotecan. However, the CT/TT genotype in the *rs3200401* MALAT1 was significantly associated with increased toxicity to irinotecan-based regimens in colorectal cancer patients and could serve as a toxicity biomarker for irinotecan-treated patients [133]. In addition, four lncRNAs, CRNDE, H19, UCA1 and HOTAIR, which are known as predictive factors for treatment sensitivity, were found to be coupled with resistance to irinotecan in colorectal cancer patients [134]. Since these mediators are directly linked to the drug resistance, they could be used to predict drug responsiveness and to be further developed into valuable biomarkers for predicting patient response to Top I-based chemotherapy.

## 4. Clinical trials involving combination of epigenetic drugs with Top I inhibitors

The development of successful combination therapies became a cornerstone of cancer research for many reasons, including the augmented efficacy in comparison to monotherapy as they address critical pathways in additive or synergistic ways. In addition, combination therapies can minimize the toxicity of the administered drugs because they offer the use of individual medications at lower dosages while maintaining therapeutic efficacy [135,136]. Epigenetic drugs have been utilized to treat various cancer types either as a standalone treatment or in combination with other anticancer drugs [13]. *In vitro* and *in vivo* investigations of epigenetic drugs in combination with Top I inhibitors are plentiful and some of them showed potentially promising outcomes and were suggested to overcome drug resistance. A phase II study of epigenetic therapy including the HDAC inhibitor magnesium valproate and the non-nucleoside DNA methylation inhibitor hydralazine was used with numerous chemotherapy drugs, including topotecan. Regardless of the tumor type, hydralazine and valproate appear to vanquish topotecan resistance. The clinical benefit observed supports the hypothesis that epigenetics drive tumor cell chemoresistance including resistance to Top I inhibitors (NCT00404508) [137,138]. Consequently, a number of clinical trials have been designed for the combination of HDAC inhibitors or DNA methylation inhibitors with Top I inhibitors in patients with solid tumors. A phase I trial has been done for testing the safety and tolerability of guadecitabine (SGI-110), an FDA approved DNA methyltransferase inhibitor, and irinotecan in individuals with metastatic colorectal cancer (mCRC) who have previously received irinotecan. Guadecitabine and irinotecan with growth factor support (GFS) were safe and tolerable in patients with mCRC, with early indication of benefit (NCT01896856) [139,140]. In addition, a phase I/II trial of valproic acid plus Karenitecin was done in patients with stage IV melanoma. The results of this trial showed that this combination was well tolerated. At the maximal tolerated dose, histone hyperacetylation was detected in peripheral blood mononuclear cells, with approximately half of the treated patients achieving disease stability (NCT00358319) [85,141].

Combination treatment involving EZH2 inhibitors and Top I inhibitors has been suggested to increase the sensitivity to Top I inhibitors. Thus, a phase I/II clinical trial has been started with an original primary outcome to evaluate the safety and efficacy of the combination of EZH2 inhibitor CPI-0209 with irinotecan in patients with advanced solid tumors and lymphomas (NCT04104776) [142]. Two clinical trials for combining EZH2 inhibitors with Top I inhibitors are still ongoing in patients with SCLC. These are phase I/II trial of DS-3201b, an EZH1/2 inhibitor, in combination with irinotecan (NCT03879798) and phase I trial of tazemetostat, an oral selective inhibitor of mutant and wild-type EZH2, in combination with topotecan and pembrolizumab (NCT05353439) [143,144]. Table 1 shows the number and types of current clinical trials comprising Top I inhibitors and epigenetic drugs that are registered on ClinicalTrials.gov [138,140–145].

**Table 1.** Clinical trials for combination of epigenetic drugs and Top I inhibitors.

Top I inhibitor	Epigenetic drug	Phase	Cancer type	Status	Identifier no.
Topotecan	Hydralazine, Magnesium valproate	II	Cervical, Breast, Lung, testicular, Ovarian	Completed	NCT00404508
Topotecan	Vorinostat	I/II	Small cell lung	Terminated (insufficient enrollment)	NCT00697476
Topotecan	Tazemetostat	I	Small cell lung	Recruiting	NCT05353439
Karenitecin	Valproic acid	I/II	Melanoma	Terminated	NCT00358319
Irinotecan	Guadecitabine	I/II	Colorectal	Completed	NCT01896856
Irinotecan	CPI-0209	I/II	Urothelial, Ovarian, Endometrial, Lymphoma, Mesothelioma, Prostate	Recruiting ‘primary outcome changed’	NCT04104776
Irinotecan	DS-3201b	I/II	Small cell lung	Recruiting	NCT03879798

## 5. Conclusions

Epigenetic events are deeply involved in determining the response of cancer cells to cancer therapeutics. A variety of gene expression patterns are produced by the high rate of epigenetic modifications in tumors, and these patterns can quickly change in response to drug therapy, resulting in the development of acquired resistance and modifications of therapeutic responses. DNA and histone modifications can regulate the response to many drugs, including Top I inhibitors. Since these modifications affect the expression of different genes as well as the pattern of replication, they can be an important determinant of the response to Top I inhibitors and may play important roles in the Top I mediated DNA damage and/or the repair processes. Noncoding RNAs including miRNAs and lncRNAs are now recognized to play important roles in maintenance of genomic expression and stability. They can be utilized as promising novel therapeutic options in regulating chemosensitivity of cancer cells to Top I inhibitors and could potentiate Top I inhibitor-induced cell death. However, as we continue to learn more about epigenetics and their various functions, the current available data should open up new avenues for research into how these mechanisms affect the replication and repair processes as well as how they interact with the response to Top I inhibitors and an array of other drugs.

## 6. Expert opinion

For decades, drug resistance towards the conventional therapeutic regimens has imposed a significant challenge for the successful management of cancer. Epigenetic modifications play a crucial role in the individual patient’s response to anticancer drugs and in drug resistance [146]. Based on the data presented here, Top I inhibitors are one of the major anticancer families affected by epigenetic alterations. DNA hypomethylation can either increase or decrease sensitivity to Top I inhibitors and this might be dependent on the location, the position of the methyl residues with respect to the cleavage site, the type of genes affected and the degree of DNA methylation [61,64,66]. The combination of HDAC inhibitors and Top I inhibitors either as independent agents or as dual inhibitors can result in enhanced cytotoxic effects, anticancer activity and sensitization of resistant cells to Top I inhibitors [78,80,84,90]. Inhibition of HATs, KDMs and EZH2 can result in enhanced efficacy and decreased resistance to Top I inhibitors [93,112,114,115]. BET inhibition can have additive to synergistic effects when combined with Top I inhibition [101,103]. These effects would be an essential strategy for enhancing the activity of Top I inhibitors in cancer cells. Recently, miRNAs and lncRNAs were suggested to be utilized as new therapeutic targets to modulate the chemosensitivity of cancer cells to Top I inhibitors. Thus, epigenetic modifications hold great potential to be used as predictors or adjunct to sensitize cancer cells to Top I inhibitors and more of them like histone phosphorylation, ubiquitylation and sumoylation might also be linked to the response to Top I inhibitors and could be investigated in the future. Furthermore, combination therapies using epigenetic drugs and Top I inhibitors together could hold the promise for effective targeted therapeutic strategies. These combination therapies might also take advantage of the use of other agents that can enhance the epigenetic drugs’ action, such as inhibitors of metabolic enzymes (e.g. UGT1A1) or inhibitors of helicases to augment the effect of the epigenetic drugs on the response to Top I inhibitors and attenuate the development of resistance. We propose that when assessing resistance to Top I targeted drugs, more consideration should be given to epigenetic alterations.

Finally, the study of epigenetics is a fast-growing area. It will help us to further explain the processes of drug response and resistance as well as advance our knowledge of different pathways involved in disease progression. Many epigenetic inhibitors are currently in clinical trials and others have already received FDA approval. However, the discovery of drugs that interfere with both epigenetic mechanisms and traditional targets of anticancer agents, as well as the use of noncoding RNA techniques to specifically target gene expression, would be the most challenging yet promising future work to be done. We believe the content of this review could be of much benefit in the evolving field of epigenetics as druggable targets and potential biomarkers for Top I and its inhibitors. It could pave the way for the implementation of epigenetics as routinely checked predictors of response to other types of drugs and could have important clinical implications that can now be explored.

## Data Availability

Data sharing not applicable – no new data generated.
